# Reentry Trajectory Optimization Based on a Multistage Pseudospectral Method

**DOI:** 10.1155/2014/878193

**Published:** 2014-01-16

**Authors:** Jiang Zhao, Rui Zhou, Xuelian Jin

**Affiliations:** ^1^School of Automation Science and Electrical Engineering, Beihang University, Beijing 100191, China; ^2^Beijing Microelectronics Technology Institute, Beijing 100076, China

## Abstract

Of the many direct numerical methods, the pseudospectral method serves as an effective tool to solve the reentry trajectory optimization for hypersonic vehicles. However, the traditional pseudospectral method is time-consuming due to large number of discretization points. For the purpose of autonomous and adaptive reentry guidance, the research herein presents a multistage trajectory control strategy based on the pseudospectral method, capable of dealing with the unexpected situations in reentry flight. The strategy typically includes two subproblems: the trajectory estimation and trajectory refining. In each processing stage, the proposed method generates a specified range of trajectory with the transition of the flight state. The full glide trajectory consists of several optimal trajectory sequences. The newly focused geographic constraints in actual flight are discussed thereafter. Numerical examples of free-space flight, target transition flight, and threat avoidance flight are used to show the feasible application of multistage pseudospectral method in reentry trajectory optimization.

## 1. Introduction

Global strike and space transportation have spurred a great interest in hypersonic glide vehicle for both military and civilian applications [1, 2]. The need for an effective and reliable access to space is promoting a rapid development of hypersonic glide vehicle. The progress is witnessed by the experimental success of NASA's scramjet-powered X-43A in 2005, US Air Force's X-51 in 2010, and the recent flight of DARPA's Falcon HTV-2 in 2012.

The reference trajectory is one of the key components of reentry guidance design for hypersonic glide vehicle; therefore, reentry trajectory optimization plays an important role in steering a safe and efficient flight of hypersonic glide vehicle in complex reentry environment, as well as meeting all of the mission requirements [3]. Generally, the hypersonic reentry vehicle enters the atmosphere of the Earth at an altitude of about 100~120 km. The full flight trajectory ranges from the high orbital reentry interface to the terminal area at 20~30 km in altitude. The reference trajectory is typically generated offline and preloaded on the hypersonic glide vehicle before launching. It is often required to correct the reentry trajectory for tracking errors during the reentry flight and even to replan a reference trajectory onboard for reaching a new target or aborting. It is a challenging task to optimize a reference trajectory in real-time for hypersonic glide vehicle, since the dynamics model is highly nonlinear along with limited control authority in the reentry flight [4].

The overall objective of this paper is to develop an onboard control strategy for reentry trajectory optimization. The multistage pseudospectral method is proposed to deal with the unexpected situations in reentry flight such as target transition and threat avoidance. In each processing stage, the trajectory estimation and trajectory refining are conducted to generate a specified range of flight trajectory. The full trajectory is determined in the form of optimal trajectory sequences. The main results on analysis of reentry trajectory optimization by using multistage pseudospectral method are presented to validate the feasibility.

## 2. Brief Review

Generally, two categories of numerical methods are used to solve the problem of reentry trajectory optimization: the indirect methods and the direct methods [5]. The indirect methods are based on the Pontryagin's minimum principle that results in a Hamiltonian boundary-value problem (HBVP). A high accuracy in the solution is the primary advantage of the indirect methods; however, the HBVP is quite complicated to solve [6]. The direct methods mainly convert the optimal control problem to the nonlinear programming problem (NLP). It is easier to use due to the larger radii of convergence without deriving the first-order necessary conditions [7].

Of the many direct methods, pseudospectral method has been demonstrated as an effective tool for solving the problem of reentry trajectory optimization. The pseudospectral method is one class of state and control parameterization methods. It was first used in optimal control problems by Reddien [8] in 1979. Recent studies have shown that the pseudospectral methods can provide simple structures and faster convergence rates for the optimal control problem with smooth and well-behaved solution [9, 10]. It is quite convenient to obtain the solutions of large-scale constrained optimal control problem in a computationally efficient manner.

The well-known pseudospectral methods mainly include the Chebyshev pseudospectral methods (CPM) [11, 12], the Legendre pseudospectral methods (LPM) [13–19], the Gauss pseudospectral methods (GPM) [20–27], and the Radau pseudospectral methods (RPM) [28–34]. The key features of all these pseudospectral methods are listed in Table 1.

1. LPM: Rao and Clarke [13] first used the LPM to optimize the reference trajectory for a high lift-to-drag ratio reentry vehicle with path constraints and control authority. The results demonstrated that the trajectory obtained by the LPM is extremely close to an optimal trajectory derived from the first-order necessary conditions. Later, Rao [14] also presented an extension of the LPM for nonsequential multiple-phase optimal control problems using the continuity conditions on the state and control.
2. The successful use of the LPM in reentry trajectory optimization promoted its update for a simple way to check the optimality of the direct methods. Gong and Ross [15] proposed the convergence results for problems with mixed state and control constraints. The research has shown that, under a set of sufficient conditions, the discretized solution converges to the continuous solution. Further, Williams [16, 17] introduced several variants of the standard LPM providing general pseudospectral approaches to find the CP. Recent studies have focused on the solutions of reentry trajectory optimization with maximum downrange [18, 19]. The feasible reentry trajectory obtained by the LPM can reach an accuracy of 10^−3^~10^−5^.
3. GPM: Benson et al. [20] first expatiated on the integral GPM and the differential GPM, explicitly formulating a mapping between the Karush-Kuhn-Tucker (KKT) conditions and the discretized first-order necessary conditions. Huntington [7] improved the GPM by a revised pseudospectral transcription for problems with path constraints and differential dynamic constraints. Huntington et al. [21] also presented a new method to compute the control at boundaries. Later, Jorris et al. [22] addressed the ability of the GPM to optimize the reentry trajectories for the hypersonic glide vehicle with highly accurate solutions. Jorris and Cobb [23] also proposed an up-and-coming numerical technique based on the GPM, capable of generating a three-dimensional reentry trajectory with geographic constraints. Zhang and Chen [24] introduced an easy GPM for optimizing the reentry trajectory of common aero vehicle (CAV) satisfying all of the path constraints and control authority. Tawfiqur et al. [25] and Xie et al. [26] obtained the flight profile using multiphase implementation of the GPM. Yang and Sun [27] improved the GPM to solve the problem of minimum total heat and demonstrated that the approach is not sensitive to the initial value.
4. RPM and CPM: The RPM and CPM remain the less studied of the pseudospectral methods; however, the two new pseudospectral methods migrate fast from theory to flight application in the last years. One of the key advantages is that the RPM provides an accurate way to construct a complete mapping between the KKT multipliers in NLP and the costates in the optimal control problem [28, 29]. Unlike the LPM, the costate approximation of the RPM converges exponentially. The RPM is comparable with the GPM in accuracy and computational efficiency, while only resulting in a less accurate final costate than the GPM [9, 10]. The RPM for solving infinite-horizon nonlinear optimal control problems was developed by Fahroo and Ross [30], followed by a research on direct trajectory optimization and costate estimation for finite-horizon problems [31]. Ross and Fahroo [32] and Huntington et al. [9] also demonstrated when the RPM probably fails and when it is appropriate to use it for optimal control problems. Recent studies have focused on the generation of optimal reentry trajectories for the RLV and suborbital launch vehicle (SLV) using the RPM and CPM [12, 33, 34].

Although pseudospectral methods have achieved numerous advances in direct trajectory optimization, a drawback of the techniques is that the process of global trajectory optimization is time-consuming, such that the reference trajectory has to be obtained before flight. It also cannot deal with the unexpected situations in the glide flight such as the target transition and threat avoidance. For the purpose of fully autonomous and adaptive reentry guidance, it is of great importance to enable the onboard trajectory control strategy, which generates the optimal or suboptimal trajectory with the transition of the flight state.

## 3. Fundamentals

### 3.1. Reentry Dynamics

Using the spherical rotating Earth model, the 3DOF point-mass dynamics of reentry vehicles are described by the following equations of motion [35]:

(1) r˙=Vsinγθ˙=(Vcosγsinψ)(rcosϕ)ϕ˙=(Vcosγcosψ)rV˙=−Dm−gsinγ +Ω2r(sinγcosϕ−cosγsinϕsinψ)cosϕγ˙=Lcosσ(mV)−gcosγV+Vcosγr+2Ωcosϕcosψ +Ω2rcosϕ(cosγcosϕ+sinψsinϕsinγ)Vψ˙=Lsinσ(mVcosγ)−Vcosγcosψtanϕr +2Ω(tanγcosϕsinψ−sinϕ) −Ω2rsinϕcosϕcosψ(Vcosγ),

where *r* is radial distance from the center of the Earth to the reentry vehicle. The terms *θ* and *ϕ* are the longitude and latitude. The Earth-relative velocity is *V*. The heading angle is *ψ*, and *γ* is the flight-path angle. The mass of the vehicle and the bank angle are described as *σ* and *m*, respectively. The terms *D* and *L* are the aerodynamic drag and lift forces; that is,  *D* = *ρV* ^2^ *C*_*D*_ *S*_ref_/2 and *L* = *ρV* ^2^ *C*_*L*_ *S*_ref_/2, where *ρ* is the atmospheric density and *S*_ref_ is the reference area of reentry vehicle. Both the drag and lift coefficients *C*_*D*_ and *C*_*L*_ depend on *α*, the angle of attack. Moreover, the Earth angular velocity and gravitational acceleration are described as *Ω* and *g*, respectively.

### 3.2. Typical Reentry Constraints

Typical path constraints for reentry trajectory optimization include [36]

(2) $$\begin{array}{c} \dot{Q}=K_{Q}\rho^{0.5}V^{3.15}\leq {\dot{Q}}_{\max}, \end{array}$$

(3) $$\begin{array}{c} q=\frac{1}{2}\rho V^{2}\leq q_{\max}, \end{array}$$

(4) $$\begin{array}{c} \frac{\sqrt{L^{2}+D^{2}}}{mg_{0}}\leq n_{L\max}, \end{array}$$

where (2) is a constraint on heating rate at a specified point on the surface of hypersonic vehicle with a normalization constant *K*_*Q*_. Constraint (3) is on the dynamic pressure that is determined by the atmosphere density and Earth-relative velocity. The total aerodynamic load constraint is described as (4). Note that these constraints are “hard” constraints, meaning that they should be within the maximum allowable values strictly.

Generally, the terminal conditions depend on different flight missions. Let subscript *f* denote the terminal state; the terminal constraints for the reentry trajectory are defined as

(5) $$\begin{array}{c} h_{f}=h_{f}^{*},\;\;\theta_{f}=\theta_{f}^{*},\;\;\phi_{f}=\phi_{f}^{*},\\ V_{f}=V_{f}^{*},\;\;\gamma_{f}=\gamma_{f}^{*},\;\;\psi_{f}=\psi_{f}^{*}, \end{array}$$

where *h* = *r* − *R*_*e*_ is the altitude from the sea level and *R*_*e*_ is the Earth radius. The mark * denotes the specified value at the final time *t*_*f*_.

In addition, the control $u={\left[\begin{array}{cc} \alpha & \sigma \end{array}\right]}^{T}$ corresponding to the state history should not exceed the system authority in terms of the maximum magnitudes and rates as follows:

(6) $$\begin{array}{c} \left|\alpha \right|\leq \alpha_{\max},\;\;\left|\dot{\alpha }\right|\leq {\dot{\alpha }}_{\max},\\ \left|\sigma \right|\leq \sigma_{\max},\;\;\left|\dot{\sigma }\right|\leq {\dot{\sigma }}_{\max}. \end{array}$$

### 3.3. Problem Formulation

Subject to the reentry dynamics, the purpose of reentry trajectory optimization for hypersonic vehicle is to find the angle of attack and bank angle, such that the objective function is a minimum (or a maximum), meanwhile satisfying all of the boundary conditions and path constraints.

Without loss of generality, the problem of reentry trajectory optimization is considered as the optimal control problem in the continuous Bolza form. Determine the control *u*(*τ*) ∈ *R* ^*m*^ and the state *x*(*τ*) ∈ *R* ^*n*^ that minimizes the objective function

(7)   J=Φ(x(τ0),t0,x(τf),tf) +tf−t02∫τ0τfg(x(τ),u(τ),τ; t0,tf)dτ

subject to the state dynamics, boundary conditions, and path constraints

(8) $$\begin{array}{c} \dot{x}\left(\tau \right)=\frac{t_{f}-t_{0}}{2}f\left(x\left(\tau \right),u\left(\tau \right),\tau ;\;t_{0},t_{f}\right)\\ \phi \left(x\left(\tau_{0}\right),t_{0},x\left(\tau_{f}\right),t_{f}\right)=0\\ C\left(x\left(\tau \right),u\left(\tau \right),\tau ;\;t_{0},t_{f}\right)\leq 0. \end{array}$$

Note that different objective functions are generally selected according to different flight missions of hypersonic vehicle, such as the minimum arriving time, minimum total heat load, maximum control margin, and maximum downrange or crossrange.

**Figure 1:**
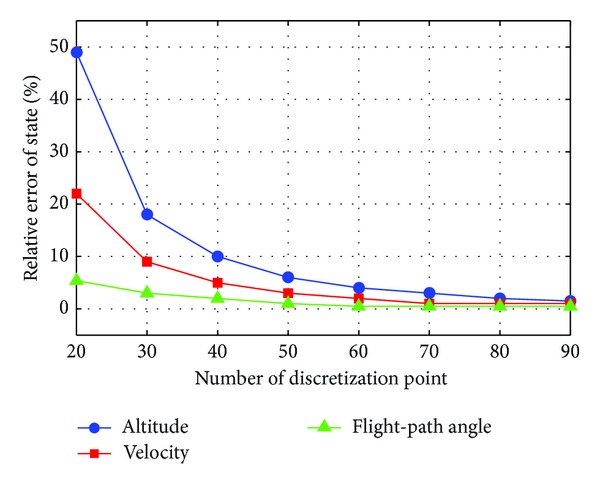
Example of relative errors between approximated and real trajectory of 25 min flight time using GPM.

The traditional pseudospectral method for solving continuous Bolza problem uses a single mesh interval and increases the degree of the polynomial for convergence [3]. It has a simple structure for the optimal control problem with smooth and well-behaved solution; however, several limitations still exist on the problem of reentry trajectory optimization. On the one hand, a fairly large-degree global polynomial is often used to obtain an accurate approximation.  Figure 1 shows an example of relative errors between approximated and real trajectory of 25 min flight time using the GPM. It can be found that, with more than 60 discretization points, the GPM typically results in a small relative error (less than 5%) between the approximated trajectory and real ODE trajectory. On the other hand, large number of discretization points probably leads to an inefficient or even intractable computation due to the large-scale global pseudospectral differentiation matrix [37].  Table 2 shows an example of computation time for optimizing a reentry trajectory of 25 min flight time using the GPM, which increases exponentially with the increasing number of discretization points.

A simple decrease of the number of discretization points would save the computation time; however, an accurate approximated trajectory is also required. A tradeoff between the computation time and accuracy of solution may not notably improve the performance of reentry trajectory optimization. In order to enable the onboard trajectory control, we add the following three objectives to the method.

1. The optimization of the specified range of flight trajectory should be completed before the hypersonic vehicle arrives at it.
2. The discretization points in the nearest interval from the present position should be dense such that the approximated trajectory is accurate enough.
3. The method is capable of dealing with unexpected situations in actual reentry flight, such as threat avoidance and target transition.

## 4. Methodology

### 4.1. Outline

In this section, we introduce the multistage pseudospectral method based on the GPM. The scheme is similar processed with the other pseudospectral methods. The traditional GPM discretizes all the state, control, and constraint condition equations at Legendre-Gauss (LG) nodes; then, it approximates the values using the Lagrange interpolating polynomials. The derivatives of each state are obtained by differentiating the global interpolating polynomials, such that the 3DOF equations of motion at the collocation nodes are transcribed into algebraic constraints. In addition, the terminal condition is defined in the forms of initial state and a Gauss quadrature. The integral parts of the objective function are also estimated by the Gauss quadrature. Thus, the continuous Bolza problem is transformed into the NLP. The optimal solution of the NLP can be obtained using the method of sequential quadratic programming (SQP).

For the purpose of onboard trajectory control strategy, the algorithm herein tactically divides the traditional GPM into multiple stages. Two subproblems are typically involved in each processing stage. One is the trajectory estimation using the low-order GPM to determine a rough global optimal trajectory. The other is the trajectory refining in the nearest interval to determine a segment of accurate trajectory. Note that the algorithm generates a specified range of trajectory at a time, ahead of the current position of vehicle. The full reentry trajectory consists of a series of optimal trajectory sequences.

In the following, the principle of GPM is briefly described. The details of the multistage trajectory control strategy and the preceding subproblems are presented thereafter. Finally, some typical geographic constraints in hypersonic glide flight are discussed.

### 4.2. GPM

The description herein is a compilation from the studies of Rao [5] and Betts and White [6]. For continuous Bolza problem, the GPM first collocates the state and control in the dynamic equation at *N* LG nodes *τ*_*k*_  (*k* = 1,2,…, *N*) that are the roots of the *N*th degree Legendre polynomial. With the two boundary nodes, *τ*_0_ and *τ*_*f*_, there are *N* + 2 discretized nodes in total. Thus, the state, *x*(*τ*), is formed with a basis of *N* + 1 Lagrange interpolating polynomials *L* as

(9) $$\begin{array}{c} x\left(\tau \right)\approx X\left(\tau \right)=\overset{N}{\underset{i=0}{\sum }}L_{i}\left(\tau \right)X\left(\tau_{i}\right)\\ L_{i}\left(\tau \right)=\overset{N}{\underset{j=0,j\neq i}{\prod }}\frac{\tau -\tau_{j}}{\tau_{i}-\tau_{j}},\;\left(i=0,1,\ldots ,N\right). \end{array}$$

Similarly, the control, *u*(*τ*), is formed with a basis of *N* Lagrange interpolating polynomials L* as

(10) $$\begin{array}{c} u\left(\tau \right)\approx U\left(\tau \right)=\overset{N}{\underset{i=1}{\sum }}L_{i}^{*}\left(\tau \right)U\left(\tau_{i}\right)\\ L_{i}^{*}\left(\tau \right)=\overset{N}{\underset{j=1,j\neq i}{\prod }}\frac{\tau -\tau_{j}}{\tau_{i}-\tau_{j}},\;\left(i=1,2,\ldots ,N\right). \end{array}$$

Then, the derivatives of each state at the LG node are described in the form of a differential approximation matrix *D* as

(11)   x˙(τk)≈X˙(τk)=∑i=0NL˙i(τk)X(τi)=∑i=0NDkiX(τi),(k=1,2,…,N).

The differentiation matrix, *D* ∈ *R* ^*N*×(*N*+1)^, is determined by

(12) Dki=L˙i(τk)={(1+τk)P˙N(τk)+PN(τk)(τk−τi)[(1+τi)P˙N(τi)+PN(τi)],i≠k(1+τk)P¨N(τi)+2P˙N(τi)2[(1+τi)P˙N(τi)+PN(τi)],i=k,

where *P*_*N*_(*τ*) is the *N*th degree Legendre polynomial [8].

Thus, the dynamic equations at the collocation nodes are transcribed into algebraic constraints as

(13) ∑i=0NDkiX(τi)−tf−t02f(X(τk),U(τk),τk; t0,tf)=0,(k=1,2,…,N).

Since the state at the final time is ignored by the state approximation equation, an additional constraint with the final state is required as

(14) X(τf)−X(τ0)  −tf−t02∑k=1Nωkf(X(τk),U(τk),τk; t0,tf)=0,

where *ω*_*k*_ are the Gauss weights.

Finally, the objective function is approximated by a Gauss quadrature as

(15)   J=Φ(X(τ0),t0,X(τf),tf) +tf−t02∑k=1Nωkg(X(τk),U(τk),τk; t0,tf)

with the boundary conditions and path constraints in the form of discretization as

(16) $$\begin{array}{c} \phi \left(X\left(\tau_{0}\right),t_{0},X\left(\tau_{f}\right),t_{f}\right)=0. \end{array}$$

(17) $$\begin{array}{c} C\left(X\left(\tau_{k}\right),U\left(\tau_{k}\right),\tau_{k};\;t_{0},t_{f}\right)\leq 0,\;\left(k=1,2,\ldots ,N\right). \end{array}$$

Thus, the solution to the continuous Bolza problem is determined by the solution to the NLP with dynamic constraints (13) and (14), objective function (15), boundary constraints (16), and path constraints (17).

### 4.3. Multistage Trajectory Control Strategy

**Figure 2:**
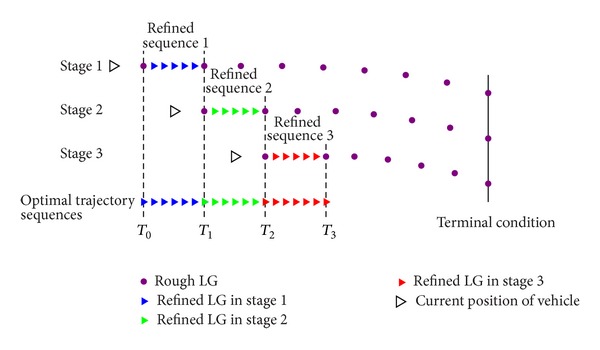
Illustration of the main idea of the multistage trajectory control strategy.

The multistage trajectory control strategy is based on the traditional GPM. The optimization problem is divided into several stages. In each stage, the algorithm solves two subproblems: the trajectory estimation and trajectory refining. The core idea of the scheme is that the collocation points should be dense enough in the interval nearest to the current position, and, in each processing stage, the method generates a specified range of reentry trajectory with the transition of the flight state.  Figure 2 is an illustration that captures the main idea of the scheme. The following steps explain the process of trajectory optimization in detail using the multistage trajectory control strategy.

**Step 1**

In Stage 1, assuming that the hypersonic vehicle is flying steadily in the current interval, the present objective is to generate a segment of accurate trajectory in the next interval. We define the next interval as *T*_0_~*T*_1_ and the trajectory segment as Sequence 1. First, the trajectory from *T*_0_ to the terminal condition of the overall trajectory is optimized by using a low-order GPM. The number of LG points in the rough optimization is *N*_rough1_ and the computation time is *T*_rough1_. Note that *N*_rough1_ is typically a small number (less than thirty) such that *T*_rough1_ is short enough. This process is called trajectory estimation. A rough optimal trajectory from *T*_0_ to the terminal condition is obtained in this step.

**Step 2**

For trajectory segment with higher accuracy, the trajectory refining is required in the nearest interval. As shown in Figure 2, the initial and terminal condition of the trajectory segment with interval *T*_0_~*T*_1_ is typically selected from among the rough optimal trajectories in Step 1. Then, the trajectory segment is optimized again by using a low-order GPM. Since Sequence 1 is a small part of the full trajectory, a low-order GPM is competent to generate an accurate trajectory segment. We define the number of LG points in the refined trajectory as *N*_refine1_ and the computation time as *T*_refine1_. Thus, the total computation time of optimization in Stage 1 is *T*_cpu1_ = *T*_rough1_ + *T*_refine1_. Sequence 1 is the optimized trajectory for the actual flight in the next interval *T*_0_~*T*_1_.

**Step 3**

When entering Stage 2, the vehicle flies along the trajectory Sequence 1 obtained in Stage 1. The next objective is to generate a segment of accurate trajectory in the interval *T*_1_~*T*_2_. The processes of trajectory estimation in Step 1 and trajectory refining in Step 2 are repeated such that the trajectory Sequence 2 can be obtained. Similarly, the numbers of LG points in the trajectory estimation and trajectory refining are *N*_rough2_ and *N*_refine2_, respectively. The total computation time of optimization in Stage 2 is *T*_cpu2_ = *T*_rough2_ + *T*_refine2_.

**Step 4**

Repeat the aforementioned processes until the vehicle gets close to the terminal condition of the full trajectory. The generation of optimal trajectory is then divided into multiple stages, and the final trajectory consists of a series of optimal trajectory sequences as shown in Figure 2.

**Figure 3:**
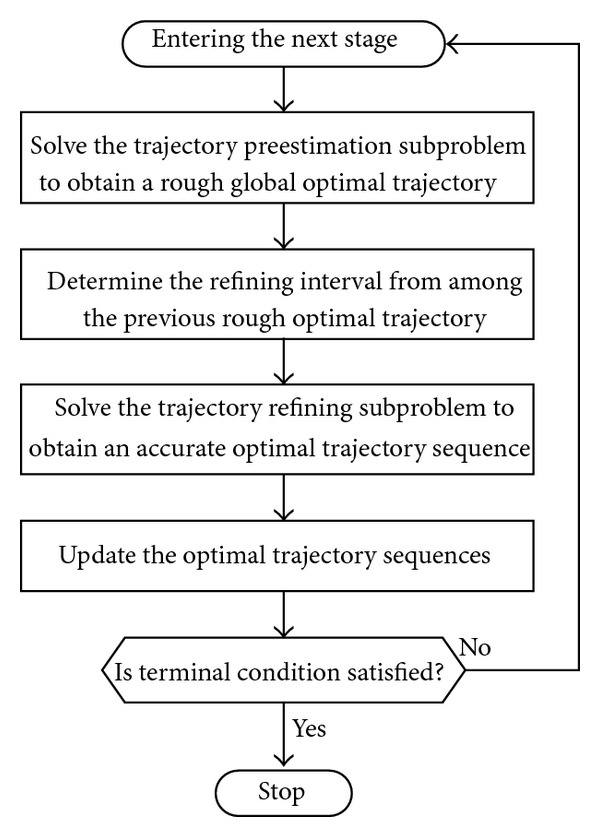
Flowchart of the multistage trajectory control strategy.

As a summary of the proposed algorithm, Figure 3 is a flowchart that captures the main blocks of the algorithm. In addition, some supplementaries of optimization process are described in the following.

1. Since the optimization algorithm is a multistage method, it typically cannot obtain the global optimal solutions. For onboard trajectory generation purpose, an optimal reentry trajectory is generally obtained in the form of local optimal trajectory sequences.
2. The choice of *N*_rough_ and *N*_refine_ is determined according to different flight missions. For the trajectory of 25 min flight time, *N*_rough_ can be selected between twenty and thirty and decreases properly as the range-to-go is reduced. For the trajectory segment with 200 sec interval *T*_*i*_~*T*_*i*+1_, *N*_refine_ is typically less than twenty.
3. The optimization of the next trajectory interval can be completed before the vehicle arrives, since the flight time in the current stage is much longer than the total computation time. For example, assuming that the trajectory interval *T*_*i*_~*T*_*i*+1_ is about 200 seconds, the optimization only needs twenty LG points for trajectory estimation and trajectory refining, respectively. The total computation time is generally less than 40~50 seconds. The flight time in each trajectory interval is 5 times more than that of the total computation time of the next trajectory interval.

### 4.4. Geographic Constraints

Since hypersonic glide vehicles take on various flight missions, some complex constraints are involved in reentry flights. The waypoints and no-fly zones are two typical geographic constraints that should be included in the reentry trajectory optimization.

For meeting the requirement of flight calibration, payload delivery, reconnaissance task, and so on, the hypersonic glide vehicle often needs to fly directly over a series of waypoints in the actual flight. Without loss of generality, the waypoint constraints are described in the form of algebraic constraints as [38]

(18) P(θ(ti),ϕ(ti))=(θ(ti)−θi)2+(ϕ(ti)−ϕi)2=0,(i=1,2,…,p),

where the position of the *i*th waypoint is presented by its longitude and latitude (*θ*_*i*_, *ϕ*_*i*_), and *p* is the total number of waypoints.

**Figure 4:**
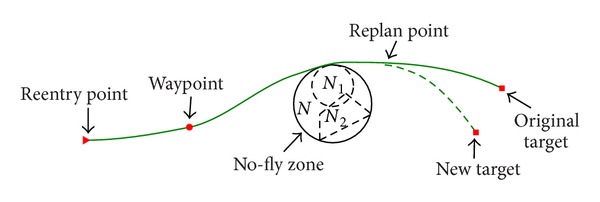
Example of the geographic constraints for glide flight.

As shown in Figure 4, a simple method to deal with the waypoint constraints is to divide the reentry trajectory into multiple phases by the waypoints. Each waypoint turns to be the last collocation node in the previous phase as well as the first collocation node in the next phase. Thus, the waypoint constraint is typically transformed into the terminal condition of the previous phase.

During the reentry flight missions, additional geopolitical sensitive regions and threat regions are also supposed to be focused on. The hypersonic glide vehicle must not violate the boundary of these regions. Without loss of generality, the no-fly zone constraints herein are specified as cylinder zones with infinite altitude, since many no-fly zones with other shapes can be replaced by the cylinder zones. As shown in Figure 4, *N*_1_ and *N*_2_ are the cross section of no-fly zones with different shape. They are convenient to be included in a larger cylinder zones of which *N* is the cross section. Thus, we can define the no-fly zones in the following form of algebraic constraints as [38]

(19) S(θ(tj),ϕ(tj))=(θ(tj)−θj)2+(ϕ(tj)−ϕj)2≥RSj2,(j=1,2,…,s),

where the cross section of the *j*th no-fly zone is described by the center (*θ*_*j*_, *ϕ*_*j*_) and the radius *R*_*Sj*_. The total number of no-fly zones is *s*.

In addition, target transition for hypersonic vehicle is another mission requirement in actual flight. In a way, the target transition is a special kind of waypoint constraints, since the new target constraint can be treated as a waypoint constraint. As shown in Figure 4, the onboard trajectory control strategy potentially plays an important role in driving the hypersonic vehicle to a new target in the presence of new mission commands or unexpected situations.

## 5. Numerical Results

In this section, we present the numerical results of reentry trajectory optimization using the multistage trajectory control strategy. The aerodynamic data and characteristics parameters are based on the CAV-H data [39, 40]. The control boundaries and hard constraint limits remain fixed throughout all the simulations as *α*_max_ = 30 deg, *α*_min_ = 5 deg, *σ*_max_ = 89 deg, *σ*_min_ = −89 deg, *Q*_*d*max_ = 8.0 × 10^5^ W/m^2^, *P*_*d*max_ = 5.0 × 10^4^ Pa, and  *n*_*L*max_ = 2.5. The objective function to find an optimal trajectory with minimum total heat load is given as

(20) $$\begin{array}{c} \min J=\int \dot{Q}\;dt. \end{array}$$

In each stage, the numbers of LG points for trajectory estimation and trajectory refining remain fixed in the following three cases as *N*_rough_ = *N*_refine_ = 20. The time interval of each refined trajectory sequence is selected around 300 seconds according to the rough trajectory estimation. The optimization solutions are found by Matlab 7.14 on a desktop computer with a 2.1-GHz processor.

### 5.1. Case 1 (Free-Space Flight)

In the numerical example of free-space flight, the initial and terminal conditions of reentry trajectory are described in Table 3.

**Figure 5:**
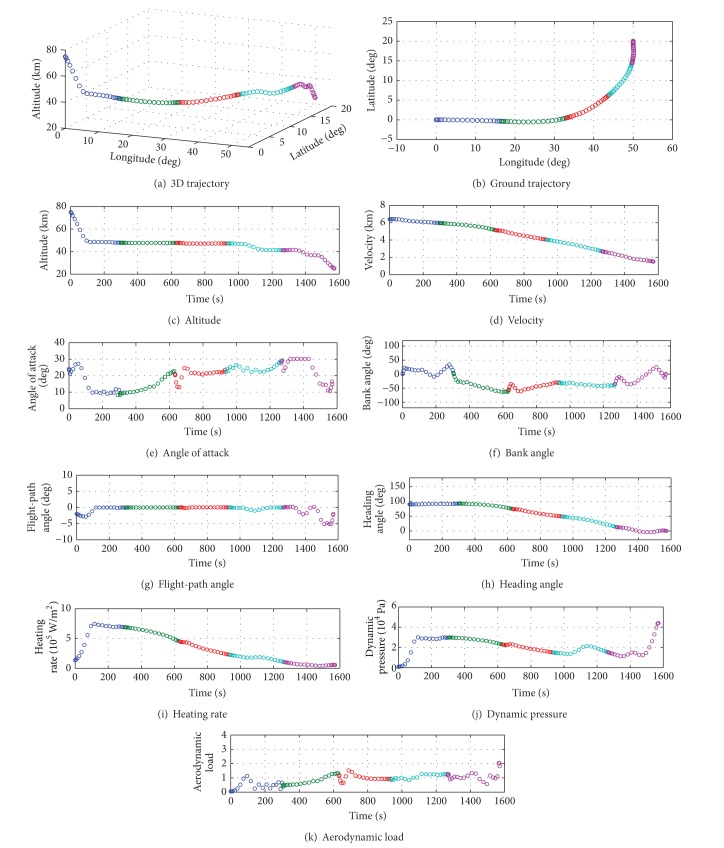
Numerical results of the free-space flight trajectory.

Figure 5 shows the numerical results of the free-space flight trajectory. The approximate values of state, control, and constraint conditions at LG nodes are represented by circle-marks. The nodes in different stage intervals are separated by different colors.

From Figure 5(a), it can be found that the 3D trajectory is smooth during all the reentry flight. The terminal conditions of the state and control are accurately satisfied as shown in Figures 5(b)–5(h). Figures 5(i)–5(k) show that the hard constraints, including heating rate, dynamic pressure, and aerodynamic load, are less than the maximum allowable values strictly. Both the angle of attack and the bank angle are also between the given boundaries of control, respectively.

The total flight time is about 1570.6 seconds. The flight time in each trajectory intervals are listed in Table 4.  Table 5 presents the computation time of the trajectory estimation and trajectory refining in each stage. It can be found that the actual flight time in the current trajectory interval is many times more than the total computation time of the next trajectory interval. All of the solution demonstrates that the multistage trajectory control strategy is feasible to solve onboard trajectory optimization problem for free-space reentry flight.

### 5.2. Case 2 (Target Transition)

In the numerical example of target transition flight, two geographic constraints are temporarily added to the flight mission when the hypersonic glide vehicle enters the second trajectory interval. The parameters of the new target and waypoint are listed in Table 6. The other conditions are the same with Case 1 as shown in Table 3.

**Figure 6:**
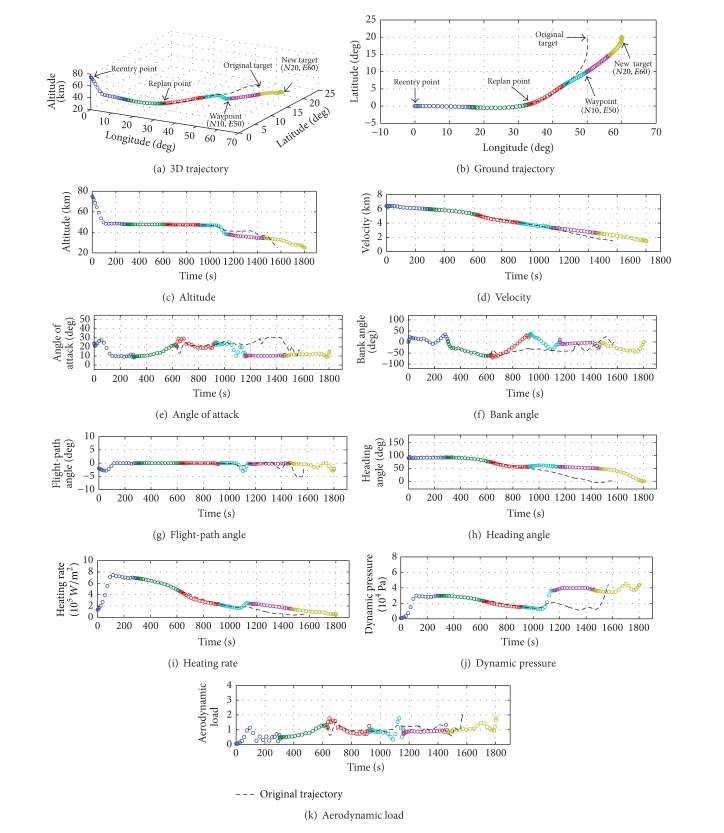
Numerical results of the target transition flight trajectory.

Figure 6 shows the numerical results of the target transition trajectory.  Figure 6(a) compares the replanned 3D trajectory with the original 3D trajectory of Case 1. It can be found that the trajectory intervals are smoothly connected around the replan point.  Figure 6(b) shows that the waypoint is directly passed through at the joint of the fourth and fifth optimal trajectory sequences. The onboard trajectory control strategy succeeds in driving the hypersonic glide vehicle to the new target. As shown in Figures 6(c)–6(k), the other terminal conditions of the state and control are also satisfied without violating the boundary of the hard constraint.

The total flight time is about 1804.6 seconds, larger than that of Case 1. The flight time in each trajectory intervals and the computation time in each stage are listed in Tables 7 and 8, respectively.

### 5.3. Case 3 (Threat Avoidance)

In the numerical example of the threat avoidance flight, one no-fly zone constraint is added to the flight mission when the hypersonic glide vehicle enters the second trajectory interval. The parameters of the no-fly zone constraint are listed in Table 9. The other conditions are the same with Case 1.

**Figure 7:**
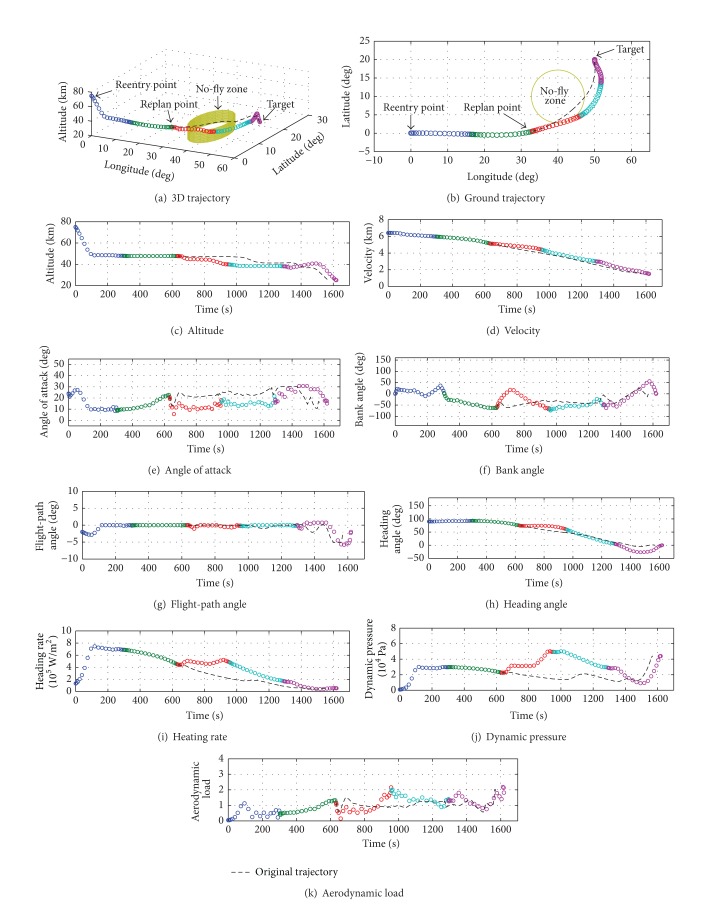
Numerical results of the threat avoidance flight.

Figure 7 shows the numerical results of the threat avoidance flight. Figures 7(a)-7(b) provide a comparison between the original trajectory and replanned trajectory. It can be found that the original trajectory would penetrate the no-fly zone directly without avoidance. In contrast, the onboard trajectory control strategy with geographic constraints succeeds in driving the trajectory to go just along the boundary of the no-fly zone. As shown in Figure 7(f), the bank angle has an enormous change compared to the original trajectory for obtaining large lateral maneuverability. Figures 7(i)-7(j) show that the heating rate and dynamic pressure increase in advance since the geographic constraint is added; however, they are less than the maximum limitations. Finally, the same target with original mission is also satisfied.

The total flight time is 1620.0 seconds. Tables 10 and 11 list the flight time and the computation time in each stage, respectively. The results demonstrate the feasibility of the multistage GPM for solving reentry flight of threat avoidance missions.

## 6. Conclusions

In this paper, a multistage trajectory control strategy based on the pseudospectral method is developed for reentry trajectory optimization. In each processing stage, the algorithm generates a specified range of optimal trajectory ahead of the current position of hypersonic vehicle. The full trajectory consists of a series of optimal trajectory sequences. Moreover, the proposed scheme is capable of dealing with unexpected situations in reentry flight. The performance of the multistage pseudospectral method is demonstrated by numerical examples of the free-space flight, target transition flight, and threat avoidance flight.

## Figures and Tables

**Table 1:** The key features of some well-known pseudospectral methods.

Methods	Interpolating polynomial	Type of CP*	Interval	Number of DP/CP*
CPM	*N* Chebyshev polynomial	Chebyshev-Gauss-Lobatto (CGL)	[−1, 1]	*N*/*N*
LPM	*N* Legendre polynomial	Legendre-Gauss-Lobatto (LGL)	[−1, 1]	*N*/*N*
RPM	*N* Legendre polynomial	Legendre-Gauss-Radau (LGR)	[−1, 1) or (−1, 1]	*N*/*N* − 1
GPM	*N* − 1 Legendre polynomial	Legendre-Gauss (LG)	(−1, 1)	*N*/*N* − 2

*DP: discretization points; CP: collocation points.

**Table 2:** Example of computation time for optimizing a reentry trajectory of 25 min flight time using GPM.

Number of discretization points	The computation time (s)
20	5~20
40	30~100
60	150~250
80	200~400

**Table 3:** Initial and terminal conditions of full trajectory (Case  1).

Conditions	*h* (km)	*V* (m/s)	*θ* (deg)	Φ (deg)	*γ* (deg)	*ψ* (deg)	*α* (deg)	*σ* (deg)
Initial	75	6400	0	0	−2	90	24	0
Terminal	25	1500	50	20	−2	0	15	0

**Table 4:** Flight time in each trajectory interval (Case  1).

Interval	*T*_0_~*T*_1_	*T*_1_~*T*_2_	*T*_2_~*T*_3_	*T*_3_~*T*_4_	*T*_4_~*T*_5_
Δ*T* (s)	304.5	327.2	305.3	330.5	303.1

**Table 5:** Computation time in each stage (Case  1).

Stage	Stage 1	Stage 2	Stage 3	Stage 4	Stage 5
*T*_rough_ (s)	22.8	16.5	15.2	16.0	18.1
*T*_refine_ (s)	17.5	14.0	14.4	13.3	—

**Table 6:** Parameters of the geographic constraints (Case  2).

Constraints	Parameter
New target	(N20, E60)
Waypoint	(N10, E50)

**Table 7:** Flight time in each trajectory interval (Case  2).

Interval	*T*_0_~*T*_1_	*T*_1_~*T*_2_	*T*_2_~*T*_3_	*T*_3_~*T*_4_	*T*_4_~*T*_5_	*T*_5_~*T*_6_
Δ*T* (s)	304.5	327.2	294.0	236.5	308.1	334.3

**Table 8:** Computation time in each stage (Case  2).

Stage	Stage 1	Stage 2	Stage 3	Stage 4	Stage 5
*T*_rough_ (s)	21.9	17.0	24.7	15.5	17.8
*T*_refine_ (s)	17.0	14.7	16.2	14.0	—

**Table 9:** Parameters of the geographic constraints (Case  3).

Constraints	Parameter
No-fly zone	Center: (N10, E50); radius: 800 km

**Table 10:** Flight time in each trajectory interval (Case  3).

Interval	*T*_0_~*T*_1_	*T*_1_~*T*_2_	*T*_2_~*T*_3_	*T*_3_~*T*_4_	*T*_4_~*T*_5_
Δ*T* (s)	304.5	327.2	323.5	337.8	327.0

**Table 11:** Computation time in each stage (Case  3).

Stage	Stage 1	Stage 2	Stage 3	Stage 4	Stage 5
*T*_rough_ (s)	22.3	18.4	21.0	17.0	18.9
*T*_refine_ (s)	16.6	16.2	15.9	16.2	—
